# Supplementary material for: Comparative Studies of Polysialic Acids Derived from Five Different Vertebrate Brains
Source: Int J Mol Sci. 2020 Nov 14;21(22):8593. doi: 10.3390/ijms21228593 (PMC7696247; doi:10.3390/ijms21228593)
Supplement: Supplementary file 1 [file ijms-21-08593-s001.zip › ijms-970131-supplementary.pptx]

## Slide 1
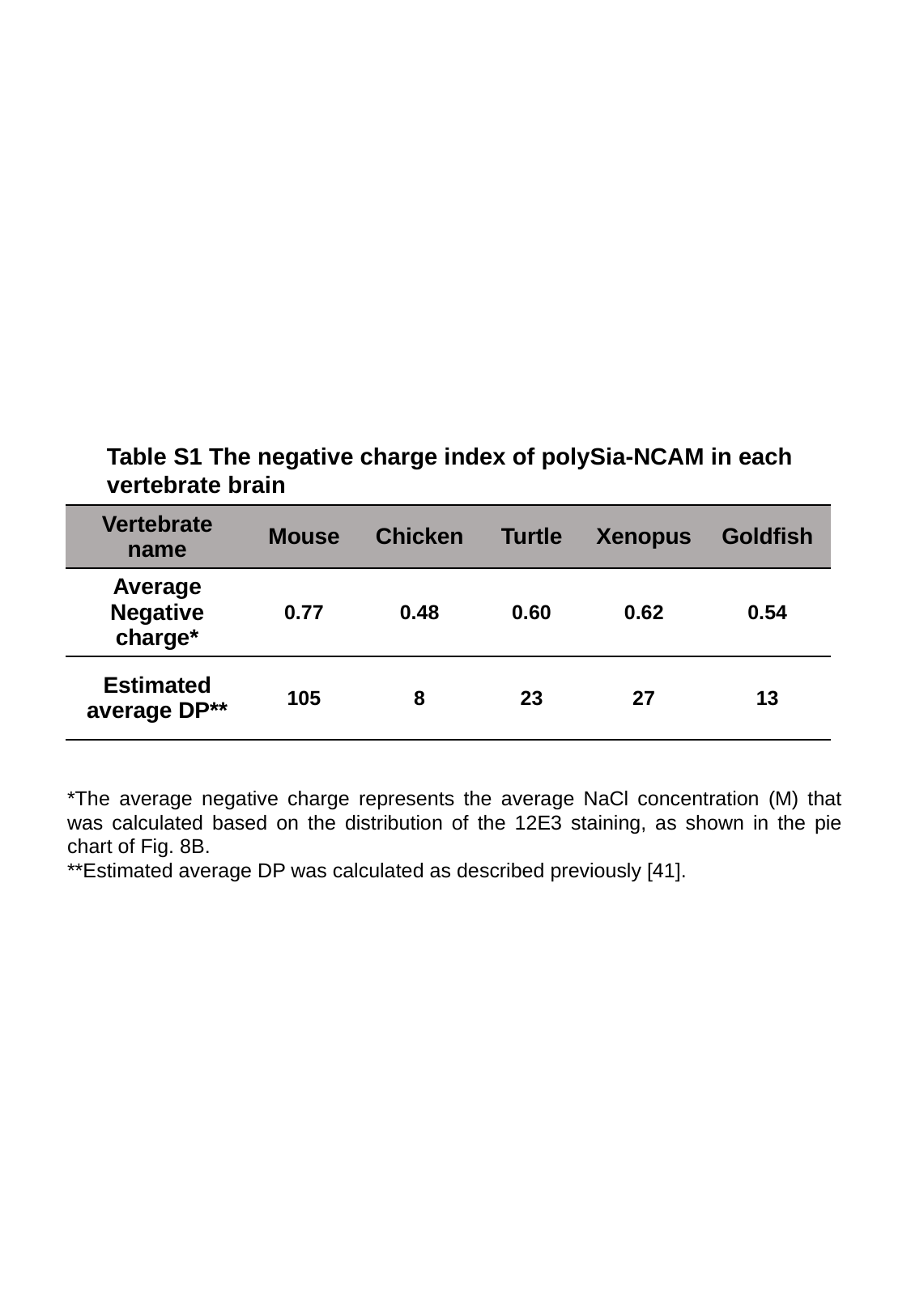

Table S1 The negative charge index of polySia-NCAM in each vertebrate brain
| Vertebrate name | Mouse | Chicken | Turtle | Xenopus | Goldfish |
| --- | --- | --- | --- | --- | --- |
| Average Negative charge\* | 0.77 | 0.48 | 0.60 | 0.62 | 0.54 |
| Estimated average DP\*\* | 105 | 8 | 23 | 27 | 13 |
*The average negative charge represents the average NaCl concentration (M) that was calculated based on the distribution of the 12E3 staining, as shown in the pie chart of Fig. 8B.
**Estimated average DP was calculated as described previously [41].

## Slide 2
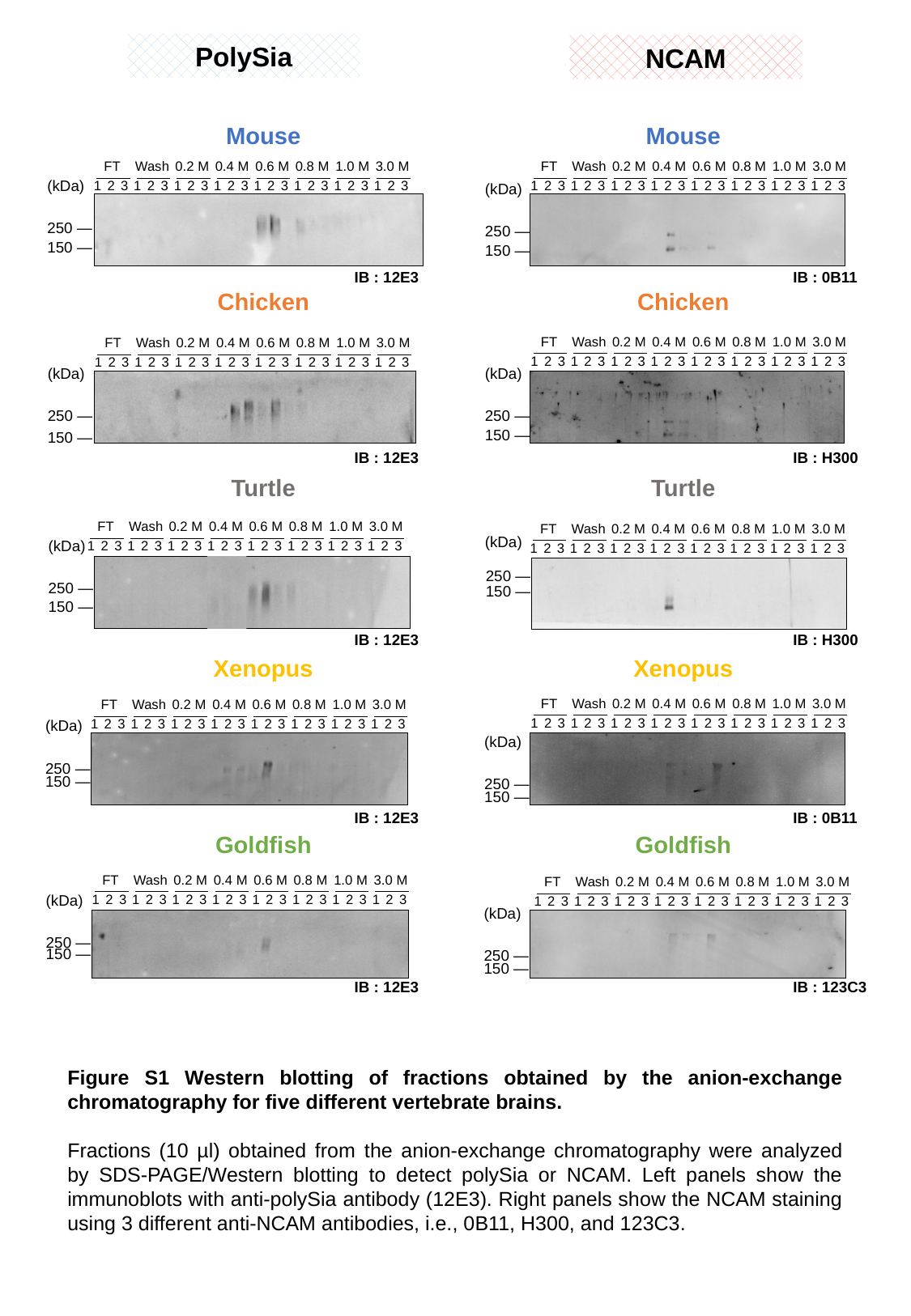

PolySia
NCAM
FT
Wash
0.2 M
0.4 M
0.6 M
0.8 M
1.0 M
3.0 M
1
2
3
1
2
3
1
2
3
1
2
3
1
2
3
1
2
3
1
2
3
1
2
3
(kDa)
250 ―
150 ―
IB : 0B11
FT
Wash
0.2 M
0.4 M
0.6 M
0.8 M
1.0 M
3.0 M
(kDa)
1
2
3
1
2
3
1
2
3
1
2
3
1
2
3
1
2
3
1
2
3
1
2
3
250 ―
150 ―
IB : 12E3
250 ―
250 ―
150 ―
150 ―
IB : 12E3
IB : H300
FT
Wash
0.2 M
0.4 M
0.6 M
0.8 M
1.0 M
3.0 M
1
2
3
1
2
3
1
2
3
1
2
3
1
2
3
1
2
3
1
2
3
1
2
3
FT
Wash
0.2 M
0.4 M
0.6 M
0.8 M
1.0 M
3.0 M
1
2
3
1
2
3
1
2
3
1
2
3
1
2
3
1
2
3
1
2
3
1
2
3
(kDa)
(kDa)
250 ―
250 ―
150 ―
150 ―
IB : 12E3
IB : H300
FT
Wash
0.2 M
0.4 M
0.6 M
0.8 M
1.0 M
3.0 M
1
2
3
1
2
3
1
2
3
1
2
3
1
2
3
1
2
3
1
2
3
1
2
3
FT
Wash
0.2 M
0.4 M
0.6 M
0.8 M
1.0 M
3.0 M
1
2
3
1
2
3
1
2
3
1
2
3
1
2
3
1
2
3
1
2
3
1
2
3
(kDa)
(kDa)
250 ―
150 ―
250 ―
150 ―
IB : 12E3
IB : 0B11
FT
Wash
0.2 M
0.4 M
0.6 M
0.8 M
1.0 M
3.0 M
1
2
3
1
2
3
1
2
3
1
2
3
1
2
3
1
2
3
1
2
3
1
2
3
FT
Wash
0.2 M
0.4 M
0.6 M
0.8 M
1.0 M
3.0 M
1
2
3
1
2
3
1
2
3
1
2
3
1
2
3
1
2
3
1
2
3
1
2
3
(kDa)
(kDa)
250 ―
150 ―
250 ―
150 ―
IB : 12E3
IB : 123C3
Mouse
Chicken
Turtle
Xenopus
Goldfish
Mouse
Chicken
Turtle
Xenopus
Goldfish
FT
Wash
0.2 M
0.4 M
0.6 M
0.8 M
1.0 M
3.0 M
1
2
3
1
2
3
1
2
3
1
2
3
1
2
3
1
2
3
1
2
3
1
2
3
FT
Wash
0.2 M
0.4 M
0.6 M
0.8 M
1.0 M
3.0 M
1
2
3
1
2
3
1
2
3
1
2
3
1
2
3
1
2
3
1
2
3
1
2
3
(kDa)
(kDa)
Figure S1 Western blotting of fractions obtained by the anion-exchange chromatography for five different vertebrate brains.
Fractions (10 µl) obtained from the anion-exchange chromatography were analyzed by SDS-PAGE/Western blotting to detect polySia or NCAM. Left panels show the immunoblots with anti-polySia antibody (12E3). Right panels show the NCAM staining using 3 different anti-NCAM antibodies, i.e., 0B11, H300, and 123C3.

## Slide 3
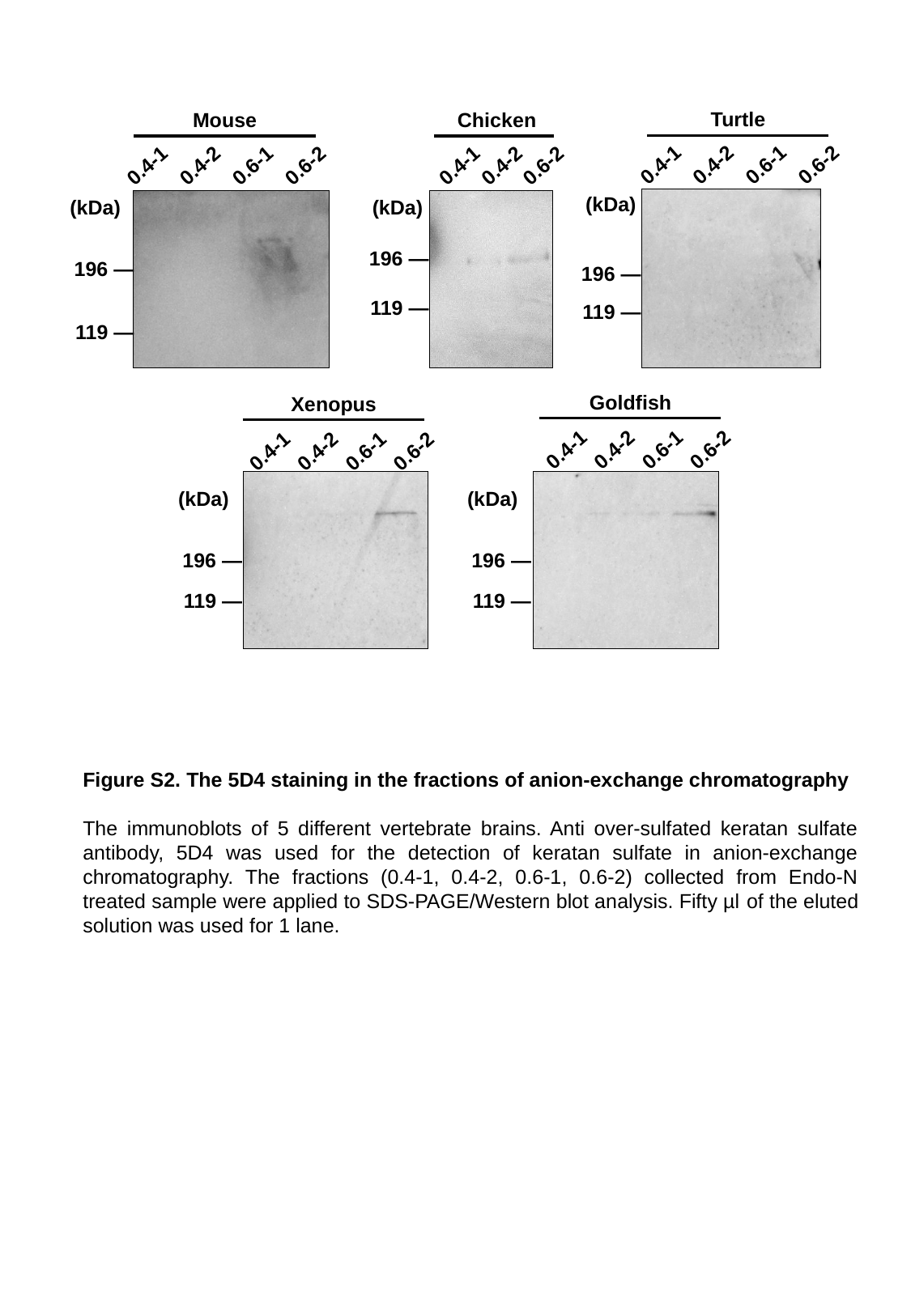

Turtle
Mouse
Chicken
0.4-1
0.4-2
0.6-1
0.6-2
0.4-2
0.4-1
0.6-2
0.4-1
0.4-2
0.6-1
0.6-2
(kDa)
(kDa)
(kDa)
196 ―
196 ―
196 ―
119 ―
119 ―
119 ―
Goldfish
0.4-1
0.4-2
0.6-1
0.6-2
Xenopus
0.4-1
0.4-2
0.6-1
0.6-2
(kDa)
(kDa)
196 ―
196 ―
119 ―
119 ―
Figure S2. The 5D4 staining in the fractions of anion-exchange chromatography
The immunoblots of 5 different vertebrate brains. Anti over-sulfated keratan sulfate antibody, 5D4 was used for the detection of keratan sulfate in anion-exchange chromatography. The fractions (0.4-1, 0.4-2, 0.6-1, 0.6-2) collected from Endo-N treated sample were applied to SDS-PAGE/Western blot analysis. Fifty µl of the eluted solution was used for 1 lane.

## Slide 4
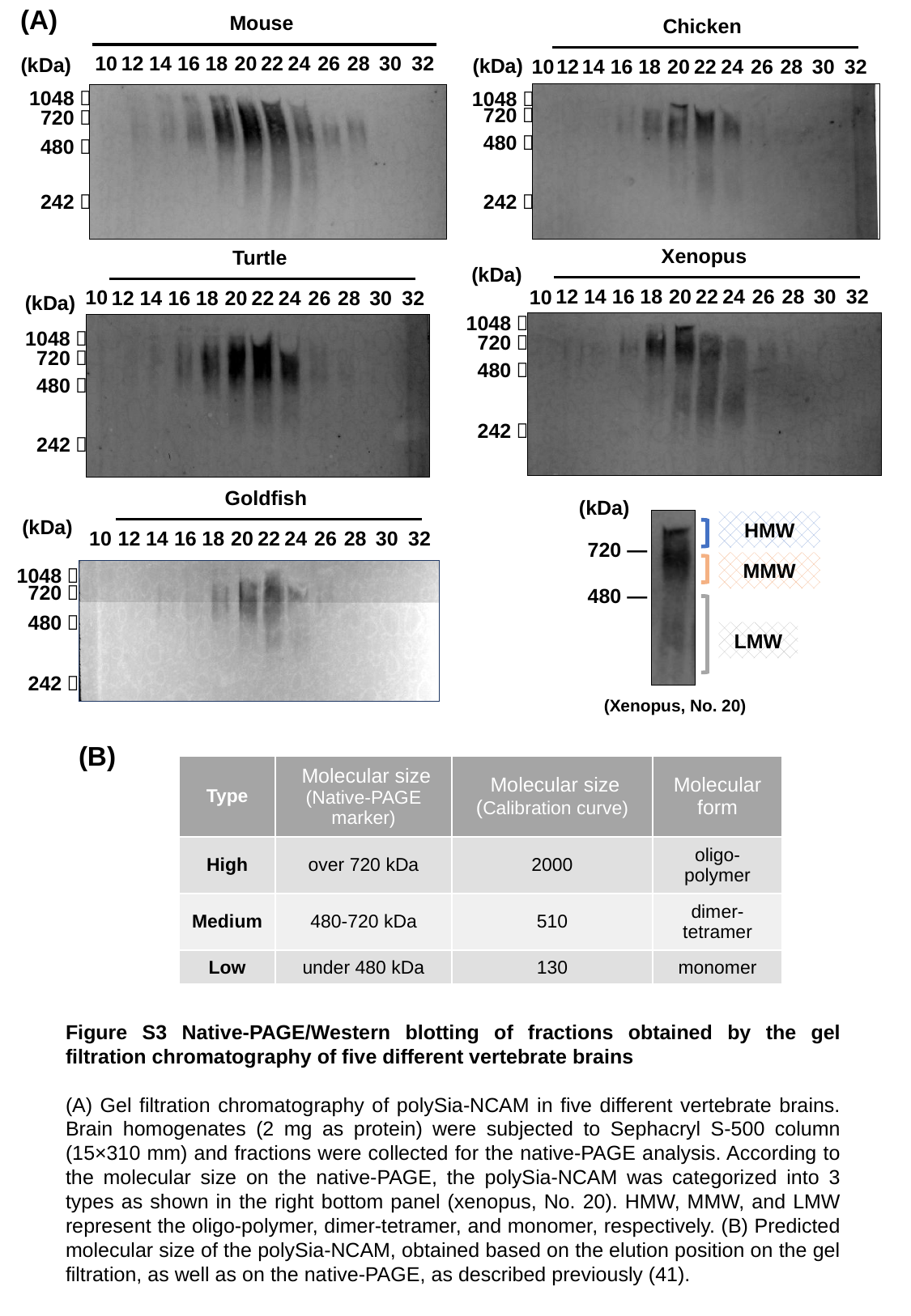

(A)
Mouse
Chicken
(kDa)
10
12
14
16
18
20
22
24
26
28
30
32
1048ー
720ー
480ー
242ー
10
12
14
16
18
20
22
24
26
28
30
32
(kDa)
1048ー
720ー
480ー
242ー
Xenopus
(kDa)
12
14
16
18
20
22
24
26
28
30
32
10
1048ー
720ー
480ー
242ー
Turtle
10
12
14
16
18
20
22
24
26
28
30
32
(kDa)
720ー
480ー
242ー
1048ー
Goldfish
(kDa)
12
14
16
18
20
22
24
26
28
30
32
10
1048ー
720ー
480ー
242ー
(kDa)
HMW
720 ―
MMW
480 ―
LMW
(Xenopus, No. 20)
(B)
| Type | Molecular size (Native-PAGE marker) | Molecular size (Calibration curve) | Molecular form |
| --- | --- | --- | --- |
| High | over 720 kDa | 2000 | oligo- polymer |
| Medium | 480-720 kDa | 510 | dimer-tetramer |
| Low | under 480 kDa | 130 | monomer |
Figure S3 Native-PAGE/Western blotting of fractions obtained by the gel filtration chromatography of five different vertebrate brains
(A) Gel filtration chromatography of polySia-NCAM in five different vertebrate brains. Brain homogenates (2 mg as protein) were subjected to Sephacryl S-500 column (15×310 mm) and fractions were collected for the native-PAGE analysis. According to the molecular size on the native-PAGE, the polySia-NCAM was categorized into 3 types as shown in the right bottom panel (xenopus, No. 20). HMW, MMW, and LMW represent the oligo-polymer, dimer-tetramer, and monomer, respectively. (B) Predicted molecular size of the polySia-NCAM, obtained based on the elution position on the gel filtration, as well as on the native-PAGE, as described previously (41).
